# The association of maternal plant-based diets and the growth of breastfed infants

**DOI:** 10.34172/hpp.2020.25

**Published:** 2020-03-30

**Authors:** Elnaz Daneshzad, Maedeh Moradi, Mohammad R Maracy, Neil R. Brett, Nick Bellissimo, Leila Azadbakht

**Affiliations:** ^1^Department of Community Nutrition, School of Nutritional Sciences and Dietetics, Tehran University of Medical Sciences, Tehran, Iran; ^2^Department of Community Nutrition, School of Nutrition and Food Science, Isfahan University of Medical Sciences, Isfahan, Iran; ^3^Department of Epidemiology & Biostatistics, School of Public Health, Isfahan University of Medical Sciences, Isfahan, Iran; ^4^School of Nutrition, Ryerson University, Toronto, Ontario, Canada; ^5^Diabetes Research Center, Endocrinology and Metabolism Clinical Sciences Institute, Tehran University of Medical Sciences, Tehran, Iran

**Keywords:** Plant-based diet, Breast feeding, Growth, Body weight, Body height, Infant

## Abstract

**Background:** Studies are needed to further understand how different plant-based dietary patterns of mothers relate to infant growth. Thus, we investigated the association between maternal plant-based diets and infant growth in breastfed infants during the first 4 months of life.

**Methods:** This cross-sectional study included 290 Iranian mothers and infants. Maternal dietary intake was assessed using a 168-question validated semi-quantitative food frequency questionnaire (FFQ). Three plant-based diet indices (PDIs) were then created to evaluate dietary intakes. Eighteen food groups were classified in three main categories by scoring method: wholeplant diet, healthy plant diet, and animal and unhealthy plant diet.

**Results:** Participants in the top tertile of unhealthy PDI (uPDI) had a lower intake of potassium,phosphorus, zinc, magnesium, calcium, folate and vitamin C, B1, B2, and B3. The upper tertileof uPDI was associated with stunting at 4-month in infants (uPDI: odds ratio [OR] = 3.27, 95%CI= 1.32, 8.10). There were no significant associations between plant-based diet scores and anthropometric indices, including weight, weight status and head circumference (P > 0.05).

**Conclusion:** In conclusion, higher adherence to uPDI may be associated with stuntingamong Iranian infants. Other PDIs were not associated with anthropometric measures. Future studies are needed to further understand the association between plant-based diets and infant growth.

## Introduction

Early childhood is a critical period for growth, and improper growth may increase the risk of chronic disease later in life.^1-3^ Stunting is a particularly widespread problem, as it is estimated to affect 30%-40% of children in low and middle income countries.^4^ A recent study revealed that linear growth failure was confined to the first months of life, which decreased by the second year of life. Stunting is found to be affected by acute insults like morbidity or inadequate feeding.^5^ Moreover, low birth weight is a significant risk factor for stunting during the first 6 months of life, therefore, prenatal intervention to optimize birth weight seems to be crucial.^6^ Breastfeeding is ideal for meeting the nutritional needs of infants,^7^ promotes growth and development and protects against infection and chronic disease.^8,9^ Thus, the World Health Organization (WHO) recommends exclusive breastfeeding for the first 6 months of life.^9^ As maternal nutrient intake influences breast milk composition, both maternal macronutrient and micronutrient intakes can influence infant growth. Micronutrients in maternal diets can influence growth through metabolic and epigenetic regulation in the offspring,^10,11^ whilst macronutrients are primarily important for energy and tissue growth.^12^ Further, maternal macronutrients imbalances, including have high-fat diets and low protein-to-carbohydrate ratios may increase the risk of obesity in infancy and childhood.^13-16^

Nutrient intake needs during lactation are well understood and the effect of maternal nutrition during lactation on infant health is generally well studied.^17^ However, less is known about how maternal plant-based diets during lactation affect infant growth. Plant based diets may be lower in important nutrients including iron, calcium, vitamin B12 and protein, but diet quality likely varies greatly based on type of plant-based diet, food preferences and socioeconomic status.^18^ It has been previously found that high meat intake paired with low vegetable intake during lactation was associated with a higher obesity risk in children when they became adolescents.^19^ Also, a smaller cohort study showed that intake of vegetables and fruit during lactation associated with breastfeeding duration, which may relate to improved health outcomes in infants.^20,21^ However, this observed association may not imply a causal relationship.^20,21^ Further differences between maternal plant-based and omnivore diet patterns, including saturated fat intake and n-6 to n-3 long chain polyunsaturated fatty acids ratio may influence growth and obesity risk of offspring.^22,23^ To the best of our knowledge, there is no study that has investigated the relationship between different maternal plant-based dietary patterns and growth of infants during lactation. Thus, the present study aimed to investigate the association between maternal plant-based diets and breast-fed infant growth, during the first 4 months of life.

## Materials and Methods

Methodology for this study adheres to Strengthening the Reporting of Observational studies in Epidemiology (STROBE) checklist.^24^ This cross-sectional study included 290 breastfeeding women and their infants, referred to health centers, after four months of exclusive breastfeeding. Sample size was calculated as follows: n= [(z_1_ -α⁄2)^2^ ×s^2^] ⁄d^2^= [(1.96^2^) × (8.2^2^)]/[0.02×60.6]^2^=177 based on the weight of participants in the Choi et al study (β = 20%, therefore the power of study is 80%).^25^ However, due to the high interest of mother’s in the study, the sample size was increased to 290 participants. Sampling was performed through multi-stage cluster random sampling. In the first stage of random sampling, all of the health centers in each of the urban geographic regions (North, South, East, West, and Central) were identified and an equal number of health centers from each region were randomly selected. An equal number of breastfeeding mothers were then randomly selected from each region. Inclusion criteria for the mother-infant dyads included non-smoking, singleton pregnancy, 4-month exclusively breastfed infant, and infant birth weight of 2500-4500 g.^26-28^ Infants were included if they were exclusively breastfed and given multivitamin drops, and not consuming any other foods, including water.^29^ Exclusion criteria included participants with diagnosed cardiovascular, liver and kidney disease and cancer, as well as mothers with a daily calorie intake of under 800 kcal/day or over 4200 kcal/day. Infants with abnormal health status, any diagnosed disease and those with micro/macrocephaly due to illnesses were excluded from the study. All mothers provided informed written consent. Following the determination of anthropometric measurements of mothers and infants, mothers filled out a demographic questionnaire as well as the International Physical Activity Questionnaire (IPAQ) and a validated food frequency questionnaire (FFQ 168 items) to assess activity and dietary intake.^26,30^ Moreover, consumption of supplements such as vitamins and minerals, milk stimulant drops or herbal and local teas intake by mothers has evaluated.

### Anthropometric and Physical Activity Assessments 

Weight and height of the mothers was measured following the International Standards for Anthropometric Assessment.^31^ Weight, height and head circumference at birth time were obtained from their mother’s delivery file. The weight, height and head circumference were measured in 2 and 4-month-year-old children by standard protocols.^31^ Specifically, weight was measured with a child’s SECA scale, height was measured with a non-plastic tape measure when children were asleep and head circumference was measured as the distance around the back of the child’s head, above the eyebrows and ears. Child growth was determined by reference growth curves for different indices such as weight for age, height for age, and head circumference for age. The WHO growth curves were used for all growth outcomes and children were categorized based on underweight (-2SD ≥ Z-score for weight for age), normal growth (-2SD ≤ Z-score for all indices ≤ +2SD), stunted (-2SD ≥ Z-score for height for age) and overweight (Z-score ≥ +2SD for weight for age) groups. Z-score of head circumference for age was used to determine microcephaly (<-2 SD) and macrocephaly of the infants (Z> +2 SD). Lastly, children > 0.67 SD for weight gain were categorized into a rapid weight gain group (WHO AnthroPlus v.1.0.4 software).

The IPAQ, assesses physical activity over the previous 7 days.^32^ There are 5 activity domains and the questionnaire assesses type, intensity and duration of activities. Physical activity of mothers was then presented as metabolic equivalent minute per week.

Socio-demographic (SES) characteristics including women and their husband’s education level and occupation, income, and home ownership status were assessed by a checklist. A score of 1 was given for high income, high level jobs, academic education and home ownership for each participant. If participants had lower income, lower level jobs, non-academic education, or were not home owners, they were given the score of 0. Scores were summed and a total SES score of 0 (low), 1 (middle class) and 2 (high) was obtained based on the tertiles of SES.

### Dietary assessment

With the help of a trained nutritionist, participants completed a validated FFQ consisting of 168 food items, describing their food intake over the past year.^26,30^ Women were included if they reported maintaining their habitual dietary pattern for the past year. Frequency of consumption of food items was considered either by day, week, month or year. The United States Department of Agriculture (USDA) household food consumption values were used for computing grams per day of each food. NUTRITIONIST-IV software (First Databank Division, The Hearst Corporation, San Bruno, CA, USA), modified for Iranian foods, was used to estimate nutrient and energy intakes.

### Plant-based diet index

Diet indexes were created similar to previous studies,^33^ including a whole plant-based diet index (PDI), healthful plant-based diet index (hPDI) and unhealthful plant-based diet index (uPDI). In this method, eighteen foods groups were created and food group consumption were computed as serving consumed per day. These food groups were on the basis of nutrient similarity. Then these 18 food groups were classified in three main categories: animal foods, healthy and unhealthy plant foods. Healthy food groups included fruits, vegetables, whole grains, legumes, vegetable oils, nuts, tea, and coffee. Unhealthy food groups included sugar-sweetened beverages, refined grains, fruit juices, potatoes, sweets, and desserts. Animal food groups included dairy, eggs, animal fats, fish and seafood, poultry, red meat and miscellaneous animal-based foods. The details of food groupings are presented in Table S1 (see Supplementary file 1). The 18 food groups were divided into quintiles of consumption, with quintiles given scores of 1 to 5. For creating PDI, the highest score (5) was dedicated to highest quintile of intake of all plant foods and the lowest score (1) was given to the lowest intakes. Participants in the highest quintile of animal foods intake were given the lowest score (1) and the highest score (5) was given to the lowest quintile of animal foods intake. Similarly, for hPDI, quintiles of healthy plant food group intakes were given scores of 1 to 5 from lowest to highest intakes. Unhealthy plant food groups and animal food groups intakes were given scores of 1 to 5 from highest intake to lowest intake. For uPDI, scores of 1 were given to the lowest quintile of less healthy plant food groups and a score of 5 given to the highest quintile, whilst for healthy plant food groups and animal food groups, scores of 1 to 5 were given from highest intake quintile to lowest intake quintile (Table S1).^34^ Finally, all 18 food group scores were summed to attain the indices score, where higher scores indicated lower animal food intake. Finally, these three indices were categorized into tertiles to assess their association with dependent measures.

### Statistical analysis

Statistical Package for the Social Sciences (SPSS) for Windows software (version 16.0, SPSS Inc., Chicago, IL) was used to analyze the data. The normality of distribution of variables, was checked by histogram and the Kolmogorov-Smirnov test. Participants were categorized based on tertiles of PDIs. Higher tertiles indicated higher compliance and lower tertiles demonstrated lower compliance. To compare the distribution of variables across the tertiles of PDIs, one-way ANOVA for continuous variables (e.g., BMI, age, weight, and height) and chi-square test for the categorical variables (e.g., socioeconomic status, herbal, milk stimulant drop and supplemental consumption) was used. Dietary intakes were compared using ANCOVA (adjusted for the energy intake of mothers). The association of maternal diet and infant growth was determined by ANCOVA (adjusted for the calorie intake of mothers) and multivariable logistic regression tests. The first model was adjusted for birth weight, the age and energy intake of mothers and the second model was additionally adjusted for BMI, physical activity, supplement intake, herbal tea consumption of mothers and SES. *P* < 0.05 was considered as statistically significant.

## Results

All 290 participants which enrolled to the study completed follow-up and included in analysis. General characteristics of mothers across tertiles of plant-based diet score types are shown in Table 1. The mean age of participants was 30.9 ± 5.2 years. Socioeconomic status did not differ based on tertiles of uPDI (*P* = 0.3). Consumption of herbal tea, breastmilk stimulant drops, and supplement consumption are low in this population (10%, 21% and 34.8%, respectively). Anthropometric characteristics of infants across tertiles of various plant-based diet score types are shown in Table S2. Dietary intakes of mothers across tertiles of PDI scores are presented in Table S3. Protein and cholesterol consumption decreased significantly across PDI, hPDI and uPDI tertiles (*P* < 0.05). Participants in the top tertiles of PDI and hPDI had a higher intake of dietary fiber, and those in the top tertile of uPDI had a lower intake of dietary fiber, compared with those in the lowest tertile (*P* < 0.05). Participants in the top tertile uPDI had a lower intake of potassium, phosphorus, zinc, magnesium, calcium, folate and vitamin C, B1, B2, and B3 compared to the lowest tertile.

**Table 1:** Demographic characteristics of mothers across tertiles of plant-based diet scores (n = 290)

**Variables**	**All**	**PDI**			***P*** *	**hPDI**			***P***	**uPDI**			***P***
		**T 1 ( ≤52.99)**	**T2 (53-57.99)**	**T3 (≥58)**		**T 1 ( ≤51.99)**	**T2 (52-57.99)**	**T3 (≥58)**		**T 1 ( ≤50.99)**	**T2 (51-58.99)**	**T3 (≥59)**	
		**n = 91**	**n = 109**	**n = 90**		**n = 88**	**n = 113**	**n = 89**		**n = 99**	**n = 96**	**n = 95**	
Age (year)^a^	30.88 (5.17)	30.69 (5.14)	31.41 (5.61)	30.43 (4.62)	0.37	30.82 (5.50)	30.81 (5.24)	31.04 (4.78)	0.93	31.56 (4.71)	30.36 (4.66)	30.71 (6.03)	0.25
Weight before pregnancy (kg)^a^	62.24 (10.08)	61.91 (10.09)	62.95 (10.75)	61.71 (9.26)	0.64	60.51 (10.65)	62.92 (8.81)	63.08 (10.88)	0.15	63.02 (9.76)	62.21 (9.38)	61.46 (11.08)	0.56
Weight after pregnancy (kg)^a^	69.27 (11.15)	68.87 (11.18)	69.88 (11.46)	68.93 (10.82)	0.77	67.34 (11.80)	70.35(10.3 0)	69.79(11.39)	0.14	69.41 (10.47)	69.27(10.67)	69.11 (12.35)	0.98
Height (cm)	161.1 (8.0)	159.8 (11.6)	162.3 (5.8)	160.9 (5.3)	0.09	161.8 (6.0)	161.8 (5.4)	159.4 (11.6)	0.06	160.8 (11.5)	161.2 (5.5)	161.3 (5.4)	0.88
Prepregnancy BMI (kg/m^2^)^a^	24.30 (7.75)	25.19 (12.66)	23.93 (4.09)	23.85 (3.43)	0.42	23.11 (3.85)	24.06 (3.45)	25.77 (12.80)	0.06	25.27 (12.12)	23.98 (3.72)	23.60 (4.02)	0.29
BMI (kg/m^2^)^a^	27.04 (8.54)	28.00 (13.91)	26.56 (4.37)	26.65 (4.11)	0.43	25.71 (4.20)	26.91 (4.05)	28.52 (14.05)	0.09	27.86 (13.35)	26.69 (4.18)	26.54 (4.46)	0.50
PA (MET min/wk)^a^	4293.2 (7386.5)	4774.9 (8775.78)	3879.2 (5110.13)	4307.7 (8194.76)	0.70	3507.4 (4407.23)	4862.6 (9433.91)	4347.3 (6758.11)	0.43	4677 (8294.64)	3562.9 (4403.65)	4631.4 (8694.10)	0.49
Socioeconomic status % (n)													
Low	40.7 (118)	26.3 (31)	42.4 (50)	31.4 (37)	0.19	33.1 (39)	41.5 (49)	25.4 (30)	0.55	33.9 (40)	29.7 (35)	36.4 (43)	0.30
Middle	55.5 (161)	35.4 (57)	35.4 (57)	29.2 (47)		28.6 (46)	36.6 (59)	34.8 (56)		35.4 (57)	36.0 (58)	28.6 (46)	
High	3.8 (11)	27.3 (3)	18.2 (2)	54.5 (6)		27.3 (3)	45.5 (5)	27.3 (3)		18.2 (2)	27.3 (3)	54.5 (6)	
Herbal tea consumption % (n)													
Yes	10 (29)	37.9 (11)	37.9 (11)	24.1 (7)	0.62	31.0 (9)	34.5 (10)	34.5 (10)	0.85	20.7 (6)	51.7 (15)	27.6 (8)	0.06
No	90 (261)	30.7 (80)	37.5 (98)	31.8 (83)		30.3 (79)	39.5 (103)	30.3 (79)		35.6 (93)	31.0 (81)	33.3 (87)	
Supplement consumption % (n)													
Yes	21 (61)	37.7 (23)	34.4 (21)	27.9 (17)	0.48	31.1 (19)	41.0 (25)	27.9 (17)	0.86	36.1 (22)	29.5 (18)	32.3 (74)	0.79
No	79 (229)	29.7 (68)	38.4 (88)	31.9 (73)		30.1 (69)	38.4 (88)	31.4 (72)		33.6 (77)	34.1 (78)	34.4 (21)	
Milk stimulant drop consumption % (n)													
Yes	34.8 (189)	32.7 (33)	40.6 (41)	26.7 (27)	0.50	26.7 (27)	40.6 (41)	32.7 (33)	0.61	39.6 (40)	30.7 (31)	29.7 (30)	0.35
No	65.2 (101)	30.7 (58)	36.0 (68)	33.3 (63)		32.3 (61)	38.1 (72)	29.6 (56)		31.2 (59)	34.4 (65)	34.4 (65)	

BMI, body mass index; PA, physical activity; PDI whole plant-based diet index; hPDI, healthful plant-based diet index; uPDI, unhealthful plant-based diet index. * P values calculated by chi-square and one-way ANOVA for qualitative and quantitative variables, respectively. ^a^ Data are mean (SD).

Anthropometric variables of infants are shown in Table S4. Twenty-six percent of children were stunted both at 2 and 4 months. Multivariable-adjusted odds ratios (ORs) for overweight and underweight stunting, micro and macrocephaly across plant-based diet tertiles in infants are reported in Tables 2 and 3 respectively. Only four infants were underweight at 4 months of age. There was no significant association between underweight at 2 months as well as overweight in children and PDIs. As is shown in Table 3, the upper tertiles of uPDI were associated with stunting at 4 months in infants (OR = 3.27, 95% CI: 1.32, 8.10). There was no association between stunting at 2 months as well as macro- and microcephaly and PDIs.

**Table 2:** Odds ratios and 95% confidence intervals for being overweight and underweight (Z score) according to tertiles of plant-based diet score among infants (n = 290)

**Variables**	**PDI**			***P*** for trend*	**hPDI**			***P*** **for trend**	**uPDI**			***P*** **for trend**
	**T1 (≤52.99)**	**T2 (53-57.99)**	**T3 (≥58)**		**T1 (≤51.99)**	**T2 (52-57.99)**	**T3 (≥58)**		**T1 (≤50.99)**	**T2 (51-58.99)**	**T3 (≥59)**	
	**n = 91**	**n = 109**	**n = 90**		**n = 88**	**n = 113**	**n = 89**		**n = 99**	**n = 96**	**n = 95**	
Overweight at 2 months (n = 46)												
Crude	1	1.10 (0.50-2.42)	1.20 (0.53-2.69)	0.65	1	1.53 (0.71-3.29)	0.89 (0.37-2.14)	0.81	1	0.75 (0.34-1.65)	0.90 (0.42-1.93)	0.78
Model 1^a^	1	1.12 (0.45-2.76)	1.39 (0.52-3.72)	0.50	1	2.56 (1.01-6.55)	1.28 (0.44-3.71)	0.66	1	0.83 (0.31-2.23)	0.69 (0.22-2.09)	0.51
Model 2^b^	1	1.33 (0.50-3.55)	1.53 (0.50-4.71)	0.45	1	2.65 (0.97-7.20)	1.40 (0.44-4.41)	0.57	1	0.87 (0.31-2.44)	0.67 (0.19-2.26)	0.51
Overweight at 4 months (n = 43)												
Crude	1	1.07 (0.46-2.49)	1.22 (0.51-2.90)	0.63	1	0.82 (0.35-1.90)	1.08 (0.46-2.52)	0.84	1	0.46 (0.19-1.09)	0.58 (0.26-1.32)	0.17
Model 1^a^	1	0.99 (0.38-2.53)	1.22 (0.44-3.37)	0.70	1	1.17 (0.45-3.05)	1.69 (0.62-4.62)	0.30	1	0.43 (0.15-1.21)	0.38 (0.12-1.20)	0.09
Model 2^b^	1	1.03 (0.36-2.89)	0.89 (0.27-2.87)	0.84	1	0. 88 (0.32-2.43)	1. 28 (0.42-3.89)	0.65	1	0. 39 (0.12-1.19)	0. 31 (0.08-1.12)	0.07
Underweight at 2 months (n = 11)												
Crude	1	1.11 (0.24-5.12)	1.36 (0.29-6.27)	0.68	1	0.37 (0.06-2.11)	1.25 (0.32-4.81)	0.70	1	0.67 (0.18-2.46)	0.16 (0.01-1.39)	0.08
Model 1^a^	1	1.38 (0.25-7.42)	1.49 (0.24-9.06)	0.99	1	0.47 (0.07-2.97)	1.05 (0.22-4.86)	0.14	1	0.32 (0.06-1.69)	0.12 (0.01-1.32)	0.47
Model 2^b^	1	1.03 (0.11-8.45)	1.46 (0.23-9.22)	0.17	1	0.33 (0.03-3.24)	1.46 (0.27-4.90)	0.56	1	0.60 (0.08-1.97)	0.27 (0.01-1.93)	0.37

PDI whole plant-based diet index; hPDI, healthful plant-based diet index; uPDI, unhealthful plant-based diet index. * *P* value was calculated by logistic regression. ^a^ Model 1: adjusted for birthday weight, age of mother, and energy intake of mother. ^b^ Model 2: model 1 + maternal BMI, physical activity, socioeconomic status, the length and number of breastfeeding, mothers macronutrients intake and infants supplemental use.

**Table 3:** Odds ratios and 95% confidence intervals for being stunted, micro and macrocephaly (Z score) according to tertiles of plant-based diet score among infants (n=290)

**Variables**	**PDI**			***P*** for trend*	**hPDI**			***P*** **for trend**	**uPDI**			***P*** **for trend**
	**T1 (≤52.99)**	**T2 (53-57.99)**	**T3 (≥58)**		**T1 (≤51.99)**	**T2 (52-57.99)**	**T3 (≥58)**		**T1 (≤50.99)**	**T2 (51-58.99)**	**T3 (≥59)**	
	**n = 91**	**n = 109**	**n = 90**		**n = 88**	**n = 113**	**n = 89**		**n = 99**	**n = 96**	**n = 95**	
Stunted at 2 months- (n=76)												
Crude	1	1.34 (0.70-2.58)	1.44 (0.73-2.82)	0.29	1	0.64 (0.34-1.21)	0.83 (0.43-1.60)	0.57	1	0.93 (0.49-1.78)	1.05 (0.56-1.99)	0.86
Model 1^a^	1	1.74 (0.73-4.15)	1.30 (0.50-3.39)	0.59	1	0.74 (0.32-1.73)	0.80 (0.33-1.93)	0.64	1	0.97 (0.40-2.32)	1.15 (0.45-2.94)	0.76
Model 2^b^	1	1.37 (0.67-2.83)	1.49 (0.65-3.42)	0.34	1	0.57 (0.28-1.16)	0.71 (0.33-1.50)	0.40	1	1.11 (0.52-2.38)	1.81 (0.77-4.28)	0.16
Stunted at 4 months- (n=76)												
Crude	1	0.89 (0.45-1.77)	2.05 (1.06-3.96)	0.02	1	0.88 (0.46-1.68)	1.10 (0.56-2.14)	0.76	1	0.89 (0.45-1.77)	2.05 (1.06-3.96)	0.02
Model 1^a^	1	0.98 (0.42-2.26)	2.23 (0.95-5.21)	0.05	1	1.27 (0.57-2.80)	1.27 (0.55-2.91)	0.57	1	1.94 (0.83-4.54)	2.86 (1.14-7.18)	0.02
Model 2^b^	1	0.83 (0.39-1.78)	2.02 (0.88-4.60)	0.08	1	0.94 (0.45-1.94)	1.05 (0.49-2.25)	0.87	1	1.79 (0.81-3.96)	3.27 (1.32-8.10)	0.01
Microcephaly at 2 months (n=11)												
Crude	1	0.40 (0.07-2.27)	1.27 (0.33-4.92)	0.68	1	0.73 (0.15-3.92)	1.68 (0.39-7.28)	0.44	1	1.75 (0.40-7.56)	1.04 (0.20-5.30)	0.95
Model 1^a^	1	0.59 (0.09-3.67)	1.72 (0.35-8.41)	0.48	1	0.84 (0.14-4.89)	1.32 (0.25-6.86)	0.71	1	1.51 (0.29-7.85)	1.27 (0.20-7.85)	0.80
Model 2^b^	1	0.59 (0.07-4.55)	0.96 (0.13-6.98)	0.96	1	0.44 (0.04-4.22)	1.02 (0.14-7.44)	0.81	1	1.40 (0.18-10.45)	0.70 (0.05-9.17)	0.77
Macrocephaly at 2 months- (n=19)												
Crude	1	0.97 (0.31-3.003)	1.01 (0.31-3.26)	0.98	1	0.63 (0.18-2.14)	1.35 (0.44-4.06)	0.55	1	0.21 (0.04-1.01)	0.92 (0.33-2.49)	0.83
Model 1^a^	1	1.006 (0.26-3.76)	1.19 (0.29-4.92)	0.79	1	0.55 (0.13-2.26)	1.22 (0.32-4.71)	0.72	1	0.31 (0.05-1.76)	1.10 (0.25-4.80)	0.83
Model 2^b^	1	1.007 (0.22-4.43)	0.93 (0.17-4.87)	0.92	1	0.43 (0.09-1.98)	1.63 (0.36-7.30)	0.54	1	0.20 (0.02-1.42)	086 (0.13-5.79)	0.98
Microcephaly at 4 months (n=15)												
Crude	1	1.70 (0.41-7.03)	2.09 (0.50-8.64)	0.31	1	0.53 (0.16-1.74)	0.40 (0.10-1.61)	0.17	1	1.58 (0.43-5.79)	1.31 (0.34-5.06)	0.69
Model 1^a^	1	1.95 (0.44-8.61)	2.05 (0.43-9.66)	0.38	1	0.63 (0.18-2.22)	0.36 (0.08-1.60)	0.17	1	1.99 (0.47-8.39)	2.16 (0.47-9.91)	0.33
Model 2^b^	1	2.35 (0.43-8.88)	2.06 (0.32-9.93)	0.48	1	0.47 (0.11-2.00)	0.35 (0.07-1.75)	0.19	1	3.14 (0.56-9.82)	2.48 (0.33-11.17)	0.40
Macrocephaly at 4 months (n=36)												
Crude	1	1.92 (0.82-4.49)	0.88 (0.32-2.41)	0.84	1	1.10 (0.46-2.61)	1.21 (0.49-2.97)	0.66	1	0.62 (0.25-1.52)	0.96 (0.42-2.17)	0.91
Model 1^a^	1	2.21 (0.91-5.37)	1.16 (0.40-3.38)	0.70	1	0.91 (0.36-2.27)	0.92 (0.34-2.44)	0.87	1	0.52 (0.19-1.38)	0.56 (0.20-1.59)	0.30
Model 2^b^	1	2.46 (0.93-6.47)	1.24 (0.38-4.04)	0.69	1	0.77 (0.30-1.99)	0.74 (0.26-2.14)	0.60	1	0.49 (0.17-1.38)	0.61 (0.19-1.99)	0.44

PDI whole plant-based diet index; hPDI, healthful plant-based diet index; uPDI, unhealthful plant-based diet index. * *P* value was calculated by logistic regression. ^a^ Model 1: adjusted for birthday weight, age of mother, and energy intake of mother. Also, we added height at birth for stunting and head circumference at birth for micro- and macrocephaly. ^b^ Model 2: model 1 + socioeconomic status, BMI and physical activity of mothers, the length and number of breastfeeding, mothers macronutrients intake and infants supplemental use.

## Discussion

To the best of our knowledge, this is the first time that the association between plant-based diet scores and anthropometric indices in infants have been reported. This cross-sectional study showed that higher adherence to uPDI was associated with a higher prevalence of stunting among 4-month-old infants. This effect was driven by unhealthy plant-based dietary patterns, as PDI and hPDI tertiles did not relate to stunting. Dietary patterns of Iranian mothers were also not related to weight status or growth outcomes in infants.

Not only is the overall dietary pattern a better indicator of diet quality than individual foods or nutrients, dietary patterns are likely also better predictors of health outcomes.^35^ Plant-based diets include foods such as vegetables and fruit, meat alternatives, dairy alternatives and grains. Greater adherence to plant-based diets associated with lower protein intake, possibly due to higher intakes of animal-based protein foods in the lower tertiles of PDI, hPDI and uPDI. It is unclear why women in the highest tertiles of hPDI and uPDI had lower energy intakes. Adherence to hPDI and uPDI may be acting as a marker of food insecurity as greater food insecurity likely results in lower intakes of animal-based foods as well as lower overall energy intakes.^36,37^ In the highest tertile of uPDI, lower intakes of nutrients including calcium, iron, fiber, folate and vitamin C are likely a result of consuming a greater amount of foods that have a lower nutrient density. This result may also highlight the ability of the diet indexes to accurately classify individuals based on food intake.

Stunting, defined as children with height for age >2 SD below the WHO median^38^ in 4-month-old infants was significantly related to greater adherence to uPDI. A total of 76 children (26.2%) were stunted at 4 months, which is similar to reports of stunting in 30%-40% of children in low and middle income countries.^4^ Based on a study by Tessema et al the prevalence of stunting was higher for infants aged 6 to 8 months (43%) compared to those in 0-5 months (26.6%) or 9-23 months (39%) category. Furthermore, mothers who did not increase their dietary intake during pregnancy and lactation were 1.6 times more likely to have a stunted child compared with mothers who increased consumption.^39^ Household food insecurity underlies hunger and malnutrition in most countries in the world.^40^ Food insecurity which is at epidemic levels in Ethiopia affecting approximately 5 million people, may lead to high level of childhood stunting as has been seen in other countries.^41^ In addition, the estimated worldwide iron deficiency in pregnant women is 42%, with a higher proportion of women being deficient in lower income countries.^42^ Though there were differences in iron intake among uPDI tertiles, the prevalence of iron deficiency is unclear as it was not assessed. Further, iron intake during pregnancy and lactation likely does not relate to linear growth.^43^ With zinc intake being lower in the highest tertiles of PDI and uPDI, it is possible that zinc relates to stunting during lactation.^43,44^ All tertiles had average zinc intakes below the Recommended Dietary Allowances of 11 mg and 12 mg for pregnancy and lactation respectively.^45^ Further, it is unclear if the small magnitude of differences in zinc intake among tertiles is enough to impair growth.

Both low protein intake and low energy intake of mothers may relate to stunting, as long term maternal nutrient intakes affects breast milk composition.^46,47^ Protein intakes were lower in the highest tertile of PDI and uDPI, meaning that less women are likely meeting the Recommended Dietary Allowance for protein during pregnancy and lactation of 1.1 g/kg/d.^48^ Protein intakes were also lower in the highest tertile of hPDI, however, the smaller magnitude of difference between tertile 1 and 3 may contribute to hPDI not relating to stunting. Energy intake in the highest tertile of uPDI was ~1000 kcal/day less than the lowest tertile. Though this may relate to stunting of infants, the relationship of energy intake of mothers and stunting in children remains unclear in this population as PDI had energy intakes ~700 kcal/day higher in the highest tertile compared to the lowest tertile. Other factors not measured may help to account for differences in stunting among tertiles including incidence and severity of illness and amount of breast milk consumed per day.^48,49^

There were no observed differences among tertiles of PDI, hPDI or uPDI for microcephaly, macrocephaly or weight status. Energy and protein intakes in infants have been shown to relate to head growth^50^ and multiple nutrients including iron,^51^ folate^52^ and vitamin B12 are important for brain development. In the current study, the lack of relationship between nutritional factors and head growth could be due to large variation in dietary intakes within a tertile or because the study was not sufficiently powered to investigate these outcomes. It is unclear why maternal dietary patterns did not relate to weight status of infants at 2 or 4 months of age. Birthweight has the potential to be an important confounder is this relationship, however, there was no association between birth weight and PDIs. Similar birth weights across tertiles may indicate the embryo grew at the expense of the mother’s body nutrient reserves. Also, with relatively few infants in overweight and underweight categories, it is possible that the study is simply underpowered to detect differences among tertiles of dietary intakes. Future longer term observational studies may be able to surmise differences in weight status among plant-based dietary tertiles when following children to school age.^53,54^

Though this study had multiple strengths including the FFQ being completed with an expert nutritionist and the ability to adjust for many potential confounding variables, this study also had some limitations. The cross-sectional nature of this study does not allow for the investigation of causal relationships. It is possible that other potential confounders were not measured in this study, such as the type of delivery of the child, biochemically assessed nutritional status of the mothers and illnesses of the infants. Though height and weight were measured, body composition was not assessed. This means that the effect of maternal plant-based diets on infant adiposity needs to be further investigated. Lastly, as this study focused on mother-infant dyads in an urban setting in Iran, results should be cautiously applied to mothers in other settings.

In conclusion, our findings revealed that higher adherence to uPDI may be associated with stunting among Iranian infants. As healthy plant-based diets were not associated with stunting, this effect was likely driven by adherence to unhealthy plant-based diets. However, to gain a longer term and more detailed understanding of the impact of plant-based diets on infant health, longer and larger prospective cohort studies, with more comprehensive outcome measures, are needed.

## Ethical approval

This study attained ethical approval by Ethical Committee of Isfahan University of Medical Sciences. Also, all participants filled a written informed consent to participate in the study.

## Competing interests

The authors declare that they have no conflict of interest.

## Funding

This study was supported by Isfahan University of Medical Sciences, Isfahan, Iran.

## Authors’ contributions

ED designed and LA supervised the study. MM and ED conducted the study. MM and MM performed the statistical analyses. ED prepared a first draft of the manuscript, and NRB, NB and LA finalized it.

## Acknowledgments

We gratefully thank the participants who took part in the present study.

## Supplementary Materials

Supplementary file 1 contains Tables S1-S4.
