# Supplementary material for: The association of maternal plant-based diets and the growth of breastfed infants
Source: Health Promot Perspect. 2020 Mar 30;10(2):152–61. doi: 10.34172/hpp.2020.25 (PMC7146038; doi:10.34172/hpp.2020.25)
Supplement: Supplementary file 1 [file hpp-10-152-s001.pdf]

Supplementary file 1

Table S1. Eighteen food groups constituting the plant-based diet score

**Healthy Food Groups**		**PDI**	**hPDI**	**uPDI**
Whole grains	Whole grain breakfast cereal and other cooked breakfast cereal, oatmeal, dark bread and Persian bread such as Sangak, brown rice, other grains, bran, wheat and popcorn	Positive scores	Positive scores	Reverse scores
Fruits	Raisins or grapes, prunes, bananas, cantaloupe, watermelon, fresh apples or pears, oranges, grapefruit, strawberries, blueberries, peaches, apricots and plums	Positive scores	Positive scores	Reverse scores
Vegetables	Tomatoes, tomato juice, tomato sauce, broccoli, cabbage, cauliflower, brussel sprouts, carrots, mixed vegetables, yellow or winter squash, eggplant or zucchini, yams or sweet potatoes, spinach, lettuce, celery, mushrooms, beets, alfalfa sprouts, garlic and corn	Positive scores	Positive scores	Reverse scores
Nuts	Nuts, pistachios, peanuts and peanut butter	Positive scores	Positive scores	Reverse scores
Legumes	String beans, soybeans, beans, lentils and peas	Positive scores	Positive scores	Reverse scores
Vegetable oils	Oil-based salad dressing, vegetable oil used for cooking, olive oil, corn oil and sunflower oil	Positive scores	Positive scores	Reverse scores
Tea & Coffee	Tea and coffee	Positive scores	Positive scores	Reverse scores
**Less healthy Food Groups**				
Fruit juices	Apple juice, orange juice, grapefruit juice and other fruit juice	Positive scores	Reverse scores	Positive scores
Refined grains	Refined grain breakfast cereal, white breads and Persian white breads such as Lavash, Barbari, Taftoon, Baget, , muffins or biscuits, white rice, crackers, pasta	Positive scores	Reverse scores	Positive scores
Potatoes	French fries, baked potatoes, potato chips	Positive scores	Reverse scores	Positive scores
Sugar-sweetened beverages	Colas with caffeine & sugar, colas without caffeine but with sugar, other carbonated beverages with sugar, non-carbonated fruit drinks with sugar	Positive scores	Reverse scores	Positive scores
Sweets and Desserts	Chocolates, candy bars, candy without chocolate, cookies (home-baked & ready-made), brownies, doughnuts, cake (home-baked & ready-made), sweet roll (home-baked & ready-made), pie (home-baked &	Positive scores	Reverse scores	Positive scores

	ready-made), jams, jellies, preserves, syrup and honey			
**Animal Food Groups**				
Animal fat	Butter added to food, butter and lard used for cooking	Reverse scores	Reverse scores	Reverse scores
Dairy	Skim low fat milk, whole milk, cream, sherbet, ice cream, yogurt, cottage cheese, cream cheese, other cheese and Persian dairy beverage (Doogh)	Reverse scores	Reverse scores	Reverse scores
Eggs	Eggs	Reverse scores	Reverse scores	Reverse scores
Fish or Seafood	Canned tuna and other canned, fresh or frozen fish	Reverse scores	Reverse scores	Reverse scores
Meat	Chicken or turkey, processed meats, liver, hamburger and beef or lamb	Reverse scores	Reverse scores	Reverse scores
Misc. animal-based foods	Pizza, mayonnaise and other creamy salad dressing	Reverse scores	Reverse scores	Reverse scores

-Positive scores indicate that participants who are in the highest quintile of a food group received a score of 5.
-Reverse scores indicate that participants who are in the lowest quintile of a food group received a score of 5.

Table S2. Anthropometric characteristics of infants across tertiles of plant-based diet scores (N=290)

Variables		All		PDI		P*		hPDI		P		uPDI		P
			T1 (≤52.99)	T2 (53-57.99)	T3 (≥58)		T1 (≤51.99)	T2 (52-57.99)	T3 (≥58)		T1 (≤50.99)	T2 (51-58.99)	T3 (≥59)	
			N= 91	N= 109	N= 90		N= 88	N= 113	N= 89		N= 99	N= 96	N= 95	
Weight (kg)														
	At birth	3.14 (0.47)	3.10 (0.50)	3.16 (0.44)	3.14 (0.47)	0.68	3.20 (0.50)	3.14 (0.41)	3.08 (0.49)	0.24	3.19 (0.40)	3.06 (0.50)	3.16 (0.49)	0.14
	2 months	5.09 (0.69)	5.13 (0.66)	5.07 (0.72)	5.09 (0.69)	0.81	5.07 (0.73)	5.14 (0.67)	5.06 (0.68)	0.68	5.16 (0.70)	4.97 (0.73)	5.15 (0.63)	0.09
	4 months	6.47 (0.79)	6.47 (0.79)	6.47 (0.86)	6.47 (0.72)	0.99	6.46 (0.85)	6.53 (0.74)	6.39 (0.81)	0.46	6.54 (0.87)	6.37 (0.76)	6.49 (0.75)	0.29
Height (cm)														
	At birth	48.9 (3.4)	49.0 (3.7)	49.1 (3.1)	48.5 (3.5)	0.44	48.8 (3.4)	49.2 (3.1)	48.7 (3.8)	0.48	48.8 (3.78)	48.9 (2.8)	49.1 (3.6)	0.79
	2 months	54.7 (4.5)	55.0 (4.4)	55.0 (4.4)	54.0 (4.6)	0.20	54.7 (4.6)	55.1 (4.2)	54.3 (4.7)	0.45	54.7 (4.7)	54.7 (3.9)	54.7 (4.9)	0.99
	4 months	59.9 (5.1)	60.1 (5.2)	60.3 (4.6)	59.0 (5.4)	0.16	60.1 (5.3)	60.3 (4.8)	59.1 (5.2)	0.24	60.0 (5.3)	59.8 (4.5)	59.7 (5.4)	0.92
Head circumference (cm)														
	At birth	35.3 (2.3)	35.2 (2.6)	35.4 (2.1)	35.2 (2.1)	0.74	35.2 (2.2)	35.3 (2.0)	35.3 (2.7)	0.89	35.5 (2.8)	35.0 (1.7)	35.3 (2.1)	0.38
	2 months	38.7 (2.6)	38.7 (3.0)	38.8 (2.4)	38.5 (2.6)	0.67	38.6 (2.3)	38.7 (2.2)	38.9 (3.4)	0.75	38.9 (3.4)	38.3 (1.5)	38.8 (2.6)	0.19
	4 months	41.7 (3.4)	41.7 (3.6)	42.0 (3.4)	41.2 (0.3)	0.31	41.5 (2.8)	41.6 (2.8)	41.8 (4.4)	0.82	41.9 (4.1)	41.3 (2.7)	41.8 (3.1)	0.39

Data are mean (SD)

* P values calculated by one-way ANOVA.

PDI: whole plant-based diet index, hPDI: healthful plant-based diet index, uPDI: unhealthful plant-based diet index.

Table S3. Nutrient intake of mothers across tertiles of plant-based diet scores (N=290)

Variables		PDI		P*		hPDI		P		uPDI		P
	T1 (≤52.99)	T2 (53-57.99)	T3 (≥58)		T1 (≤51.99)	T2 (52-57.99)	T3 (≥58)		T1 (≤50.99)	T2 (51-58.99)	T3 (≥59)	
	N= 91	N= 109	N= 90		N= 88	N= 113	N= 89		N= 99	N= 96	N= 95	
Energy (kcal)	1888.7 (638.22)	2258.9 (810.11)	2589.9 (839.42)	**<0.001**	2592.7 (868.28)	2147.4 (723.32)	2026.6 (771.49)	**<0.001**	2808 (805.98)	2156.9 (609.32)	1748.7 (636.33)	**<0.001**
Carbohydrate (g)	294.40 (87.82)	313.80 (77.61)	333.70 (87.99)	**<0.001**	303.4 (92.24)	316.3 (79.31)	321.3 (90.03)	0.051	312 (94.29)	318.9 (86.63)	310.8 (93.95)	0.471
Protein (g)	90.88 (32.16)	84.94 (28.42)	76.50 (32.33)	**<0.001**	87.90 (33.69)	84.39 (28.93)	80.24 (33.01)	**0.020**	91.68 (33.01)	84.99 (30.29)	75.55 (32.84)	**<0.001**
Fat (g)	89.11 (40.33)	83.08 (35.57)	78.43 (40.33)	**0.009**	86.87 (41.35)	81.63 (35.40)	82.63 (40.33)	0.251	83.01 (41.86)	80.55 (38.46)	87.08 (41.69)	0.142
Saturated fatty acid (g)	28.82 (13.61)	25.72 (12.08)	21.17 (13.78)	**<0.001**	28.03 (14.29)	25.27 (12.25)	22.58 (13.95)	**<0.001**	26.48 (14.97)	24.18 (13.78)	25.15 (14.97)	0.175
Cholesterol (mg)	332.80 (206.11)	264.60 (182.11)	210.00 (206.62)	**<0.001**	330.7 (208.49)	268.8 (179.22)	208.4 (203.89)	**<0.001**	307.9 (223.47)	266.7 (205.26)	230.9 (222.62)	**0.001**
Dietary fiber (g)	6.83 (4.59)	7.71 (4.08)	8.29 (4.59)	**0.001**	6.59 (4.59)	7.58 (3.91)	8.66 (4.42)	**<0.001**	8.63 (4.59)	7.69 (4.25)	6.48 (4.59)	**<0.001**
Sodium (mg)	8041 (7521.11)	8109 (6649.70)	7909 (7543.21)	0.941	8015 (7587.85)	8439 (6523.42)	7511 (7415.95)	0.271	7943 (7735.76)	8099 (7106.36)	8038 (7709.71)	0.961
Potassium (mg)	4397 (1771.70)	4438 (1566.61)	4523 (1776.81)	0.692	4216 (1773.48)	4504 (1524.65)	4618 (1733.31)	**0.020**	4837 (1723.10)	4541 (1583.03)	3958 (1717.31)	**<0.001**
Phosphorus (mg)	1922 (722.60)	1836 (638.90)	1687 (724.80)	**0.001**	1836 (747.01)	1849 (642.33)	1756 (730.15)	0.222	1929 (744.62)	1831 (684.03)	1686 (742.24)	**0.001**
Zinc (mg)	10.1 (3.91)	9.7 (3.41)	8.6 (3.91)	**<0.001**	9.47 (4.08)	9.66 (3.57)	9.35 (4.08)	0.603	10.54 (3.91)	9.60 (3.57)	8.34 (3.91)	**<0.001**
Magnesium (mg)	356.9 (134.20)	359.4 (118.70)	354 (134.70)	0.885	329.6 (131.56)	360.7 (113.01)	379.2 (128.50)	**<0.001**	396.4 (126.79)	359.2 (116.41)	313.5 (126.45)	**<0.001**
Calcium (mg)	1559 (858.80)	1469 (759.26)	1306 (861.21)	**0.002**	1438 (883.84)	1501 (759.94)	1385 (863.76)	0.222	1593 (889.46)	1474 (817.13)	1323 (886.40)	**0.010**
Iron (mg)	11.70 (0.29)	12.34 (0.26)	12.59 (0.30)	0.106	12.02 (0.30)	12.01 (0.26)	12.68 (0.29)	0.178	12.99 (0.30)	12.17 (0.28)	11.45 (0.30)	**0.005**
Folate (mcg)	321 (153.08)	329.3 (133.94)	351.8 (151.98)	**0.040**	314.8 (153.01)	335.2 (131.56)	350.5 (149.60)	**0.02**	369.5 (148.9)	336.9 (136.84)	293.2 (148.41)	**<0.001**
Iodine (Ug)	0.17 (0.05)	0.31 (0.04)	0.37 (0.05)	**0.030**	0.21 (0.05)	0.27 (0.04)	0.38 (0.05)	0.081	0.24 (0.05)	0.30 (0.05)	0.33 (0.05)	0.555
Vitamin E (mg)	14.61 (18.04)	13.49 (15.99)	13.58 (18.04)	0.701	12.63 (18.21)	14.37 (15.65)	14.46 (17.87)	0.391	13.61 (18.67)	13.99 (17.15)	14.02 (18.60)	0.961
Vitamin C (mg)	244.3 (273.51)	269.7 (241.85)	294.5 (274.36)	0.102	223.4 (272.49)	273 (234.36)	310.4 (266.36)	**0.001**	316.5 (276.57)	267.9 (254.10)	221.9 (275.72)	**0.001**

Vitamin B1 (mg)	1.41 (0.51)	1.45 (0.34)	1.48 (0.51)	0.283	1.37 (0.51)	1.46 (0.34)	1.51 (0.34)	**0.007**	1.56 (0.51)	1.44 (0.39)	1.35 (0.51)	**<0.001**
Vitamin B2 (mg)	2.77 (1.02)	2.62 (0.85)	2.41 (1.02)	**0.001**	2.62 (1.02)	2.67 (0.85)	2.51 (1.02)	0.176	2.80 (1.12)	2.64 (1.03)	2.36 (1.12)	**<0.001**
Vitamin B3 (mg)	15.39 (7.65)	15.51 (6.8)	14.94 (7.65)	0.627	15.59 (7.65)	15.11 (6.63)	15.24 (7.48)	0.736	16.27 (7.65)	15.30 (7.14)	14.28 (7.65)	**0.010**
Vitamin B12 (mcg)	9.78 (16.50)	9.53 (14.46)	5.95 (16.50)	**0.009**	9.56 (16.84)	8.67 (14.46)	7.23 (16.50)	0.255	9.78 (17.19)	8.34 (15.65)	7.31 (17.15)	0.270

All values are mean (SD) and adjusted for total energy intake. PDI: whole plant-based diet index, hPDI: healthful plant-based diet index, uPDI: unhealthful plant-based diet index.
* Calculated by multivariate analysis of variance.

Table S4. Distribution of anthropometric status in infants (n= 290)

**Variable**	**Number**	**Percent**
Underweight at 2 months of age	11	3.8
Underweight at 4 months of age	4	1.4
Overweight at 2 months of age	46	15.8
Overweight at 4 months of age	43	14.7
Stunting at 2 months of age	76	26.0
Stunting at 4 months of age	76	26
Microcephaly at 2 months of age	11	3.8
Microcephaly at 4 months of age	15	5.1
Macrocephaly at 2 months of age	19	6.5
Macrocephaly at 4 months of age	36	12.3

World Health Organization growth curves were used for all growth outcomes and growth categories were defined as underweight (-2SD≥ Z-score for weight for age), overweight (Z-score≥ +2SD for weight for age) and stunted (-2SD≥ Z-score for height for age). Z-score of head circumference for age were used to determine microcephaly (<-2 SD) and macrocephaly of the infants (Z> +2 SD).
